# Comprehensive mapping of B lymphocyte immune dysfunction in idiopathic nephrotic syndrome children

**DOI:** 10.1002/ctm2.1177

**Published:** 2023-02-05

**Authors:** Qing Ye, Huihui Liu, Dongjie Wang, Jianhua Mao

**Affiliations:** ^1^ Department of Clinical Laboratory, The Children's Hospital, Zhejiang University School of Medicine, National Clinical Research Center for Child Health National Children's Regional Medical Center Hangzhou China; ^2^ Department of Nephrology, The Children's Hospital, Zhejiang University School of Medicine, National Clinical Research Center for Child Health National Children's Regional Medical Center Hangzhou China

Dear Editor,

A potential role of B lymphocytes in idiopathic nephrotic syndrome (INS) pathogenesis has been recently identified. By cytometry by time of flight (CyTOF), our study demonstrated that the intracellular immune profile of B lymphocytes in INS patients was obviously different from that in healthy children.

The anti‐CD20 monoclonal antibody has been proven to be effective in refractory INS.^1^, ^2^ In addition, our previous studies found that at least 66% of INS children had podocyte autoantibodies. These podocyte autoantibodies are positively correlated with proteinuria, and their titer decreases rapidly after effective treatment.^3^, ^4^ Considering that B lymphocytes exert an immune response in INS patients, we performed CyTOF analyses including 11 peripheral blood mononuclear cell samples from INS children by a prospectively designated cohort to profile specific immune signatures of B lymphocytes in INS patients (Table S1). The two predefined marker panels (Panels A and B) were designed to characterize the composition and function of B lymphocytes (Tables S2 and S3).

Based on phenotype marker expression levels in B cells (Panel A), B lymphocytes were partitioned into 12 clusters. There were no variances in frequency or marker expression between the nephrotic syndrome (NS) and normal control (NC) groups (Figure 1). Meanwhile, no significant differences were found in the frequencies among the steroid‐sensitive NS before treatment (Pre‐SSNS), steroid‐resistant NS before treatment (Pre‐SRNS) and NC groups. The marker expression of each group was compared in pairs and thereafter, markers of significant differences in expression were summarized by the histogram (Figure 2). Compared with the NC group, nine of 12 clusters showed significantly high expression of CD86 in the Pre‐SSNS group, whereas this result was not obtained in the Pre‐SRNS group. Compared with the Pre‐SRNS group, nine of 12 clusters showed significantly high expression of granzyme B in the Pre‐SSNS group. As shown in Figure 2C, CD86 expression presented an increase in the kidney biopsy specimen of an SSNS patient compared with that of an SRNS patient. Overall, although there were no variances in frequency and marker expression between the NS and NC groups based on the phenotype markers, the expression levels of CD86 and granzyme B on the B‐cell surface in the Pre‐SSNS group were significantly higher than those in the Pre‐SRNS group.

**FIGURE 1:**
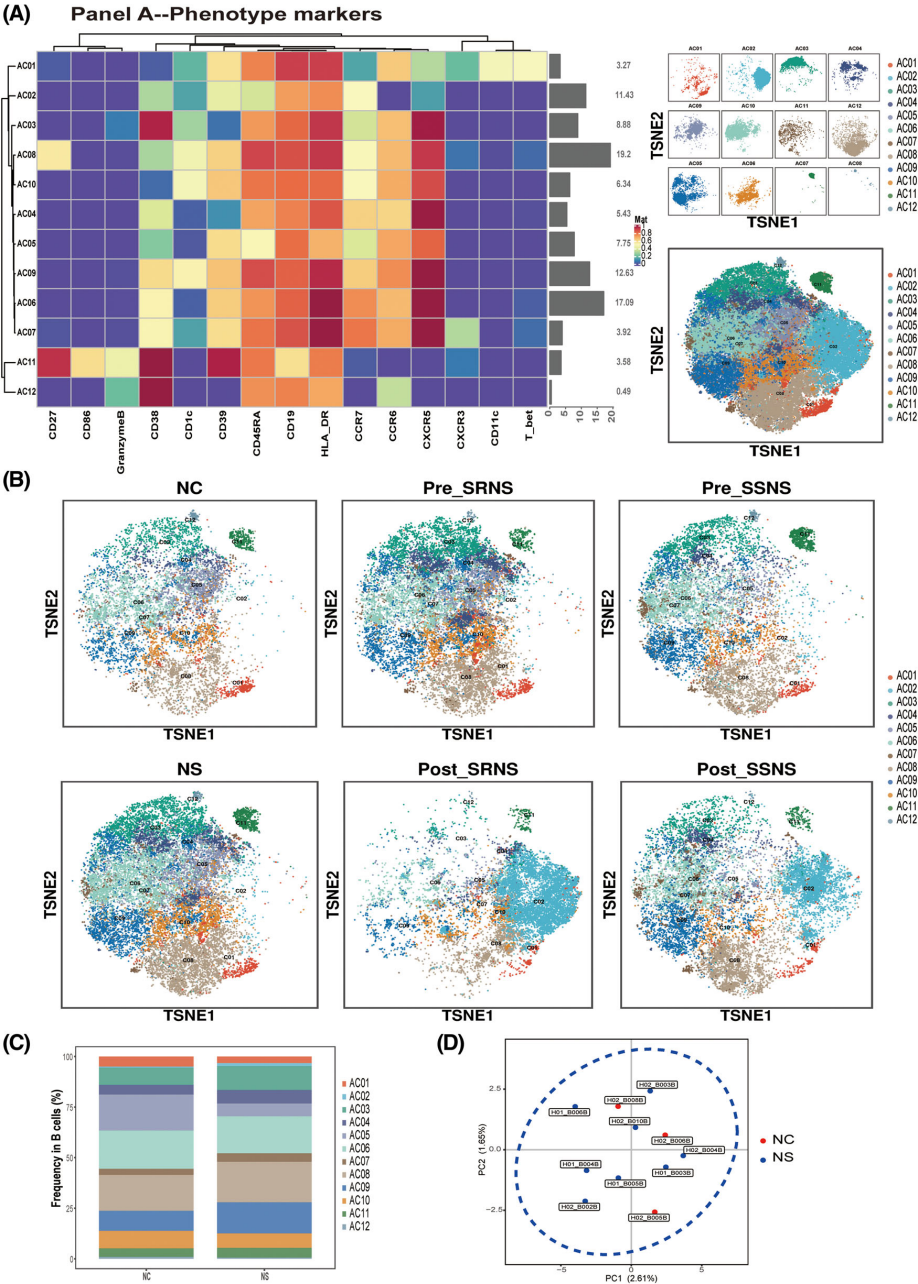
B lymphocytes were partitioned into 12 clusters based on phenotype marker expression levels (Panel A). (A) Heatmap of marker expression in each cluster. (B) The t‐distributed stochastic neighbor embedding (tSNE) plot of B cells for the six groups. (C) Comparison of frequency between the NS and NC groups by the bar plot. (D) Comparison of marker expression between the NS and NC groups by principal component analysis (PCA)

**FIGURE 2:**
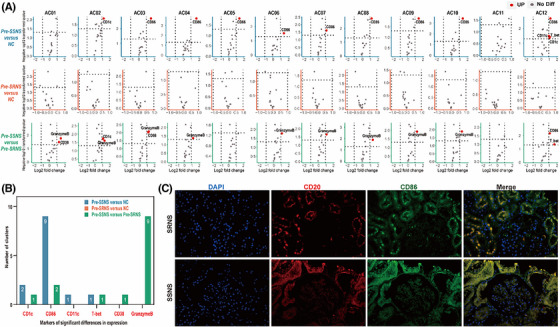
Comparison of frequency and marker expression among the SSNS, SRNS and NC groups before treatment based on phenotype markers. (A) Volcano plots present marker expression of each cluster in pre‐SSNS versus NC, pre‐SRNS versus NC and pre‐SSNS versus pre‐SRNS. Red dots represent markedly elevated marker expression in the former compared with the latter. Gray dots represent no marked differences in marker expression between the two groups. (B) The results from volcano plots are summarized by the bar plot. (C) Fluorescent images show the expression levels of CD20 and CD86 in the kidney biopsies of idiopathic nephrotic syndrome (INS) patients

After stimulation of B cells, functional marker expression levels (Panel B) were detected. There were obvious variances in frequency and marker expression between the NS and NC groups (Figure 3). The NS group exhibited a markedly higher frequency of the BC04, BC06 and BC11 clusters and a markedly lower frequency of the BC03 cluster compared with the NC group (Figure 3E). Compared with other clusters, BC03 was the only one with high expression of CD107a. Further analysis showed that the frequency and CD107a expression of CD107a^+^ B cells in the NS group were significantly lower than those in the NC group (Figure 4G). Therefore, the frequency and function of CD107a^+^ B cells were markedly suppressed in the NS group compared with the NC group.

**FIGURE 3:**
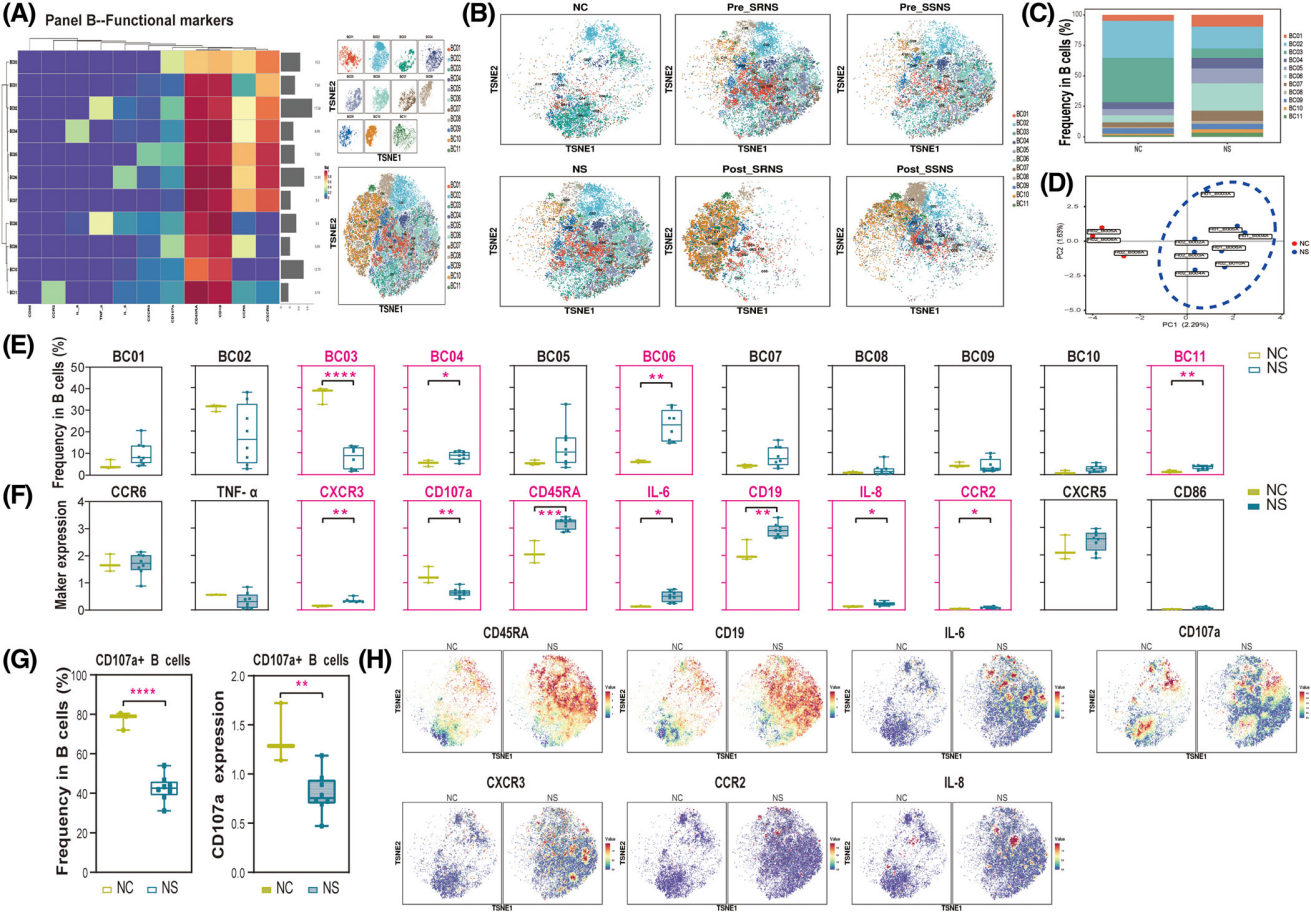
After stimulation, B lymphocytes were partitioned into 11 clusters based on functional marker expression levels (Panel B). (A) Heatmap of marker expression in each cluster. (B) The tSNE plot of B cells for the six groups. (C) Comparison of frequency between the NS and NC groups by the bar plot. (D) Comparison of marker expression between the NS and NC groups by PCA. (E) The box plots present the frequencies of each cluster between the NS and NC groups. The reddish red box represents the cluster with significant differences. (F) The box plots present the expression of each marker between the NS and NC groups. The reddish red box represents the marker with significant differences. (G) The frequency of CD107a^+^ B cells in the NS group was significantly lower than that in the NC group. The CD107a expression of CD107a^+^ B cells in the NS group was significantly lower than that in the NC group. (H) The tSNE plots of markers with significant differences between the NS and NC groups

**FIGURE 4:**
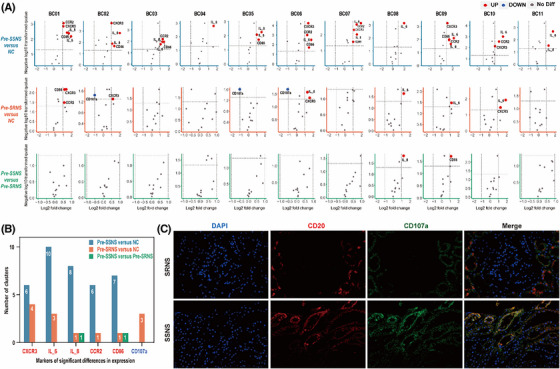
Comparison of marker expression among the SSNS, SRNS and NC groups before treatment based on functional markers. (A) Volcano plots present marker expression of each cluster in pre‐SSNS versus NC, pre‐SRNS versus NC and pre‐SSNS versus pre‐SRNS. Red dots represent markedly elevated marker expression in the former compared with the latter. The blue dot represents the marked decrease in marker expression in the former compared to the latter. Grey dots represent no marked differences in marker expression between the two groups. (B) The results from volcano plots are summarized by the bar plot. (C) Fluorescent images show the expression levels of CD20 and CD107a in the kidney biopsies of idiopathic nephrotic syndrome (INS) patients

To further explore the differences between SSNS and SRNS patients before treatment, their intracellular functional markers were analyzed again. The frequencies of BC03, BC04, BC06 and BC11 exhibited significant differences between the other groups and the NC group (Figure S1). More differences were found in Pre‐SSNS versus NC compared with the other two groups. Compared with the NC group, the expressions of interleukin (IL)‐6, IL‐8, CCR2, CXCR3 and CD86 were significantly increased in the majority of clusters and CD107a expression was not significantly decreased in the Pre‐SSNS group. However, compared with the NC group, the high expression of IL‐6, IL‐8, CCR2, CXCR3 and CD86 in the Pre‐SRNS group only occurred in a few clusters and specifically, the expression of CD107a decreased in three clusters. In addition, IL‐8 and CD86 expressions in the Pre‐SSNS group were higher than that in the Pre‐SRNS group (Figure 4B). As shown in Figure 4C, CD107a expression was decreased in the kidney biopsy specimen of an SRNS patient compared with that of an SSNS patient. Obviously, the differences in the intracellular immune characteristics of B cells between SSNS patients and healthy controls were more obvious than those between SRNS patients and healthy controls. The low expression of CD107a in B cells mainly occurred in SRNS patients.

After steroid and/or immunosuppressant treatment, the intracellular immune characteristics of B cells were similar in SSNS and SRNS patients. The frequency and CXCR5 expression of CXCR5^+^ B lymphocytes sharply decreased in SRNS patients after treatment compared with before treatment. CXCR5 expression was significantly lower in SSNS patients after treatment than before treatment (Figures S2 and S3).

Previous preclinical and clinical studies largely supported that INS was a T‐cell mediated disease.^5^ ^‐–^ ^7^ However, our study demonstrated that there were also significant changes in B cells in INS children. On the B‐cell surface, CD86 and granzyme B expression levels in SSNS patients were significantly higher than those in SRNS patients, which may provide a clue to predict steroid responsiveness.^8^ More importantly, the intracellular immune profile of B lymphocytes in INS patients was obviously different from that in healthy children. Its differences between SSNS patients and healthy controls were more obvious than those between SRNS patients and healthy controls. The frequency and function of CD107a^+^ B lymphocytes were markedly suppressed in INS patients, especially SRNS patients, compared with healthy controls. Previous research by our team has found that the diversity of T‐cell receptors (TCRs) and B‐cell receptors (BCRs) in the peripheral blood of INS patients is significantly lower than that of healthy controls by high‐throughput sequencing. Therefore, we speculate that in SRNS patients, the downregulation of CD107a^+^ B lymphocytes may give rise to the obstacle of BCR polarization, preventing B cells from presenting antigen to T cells and resulting in a decrease in TCR diversity.^9^, ^10^

In summary, we found that the intracellular immune profile of B lymphocytes in INS patients was obviously different from that in healthy children. The expression of CD86 and granzyme B on the B‐cell surface may provide a clue to predict steroid responsiveness. The frequency and function of CD107a^+^ B lymphocytes were markedly suppressed in INS patients.

## CONFLICT OF INTEREST

The authors declare that they have no conflict of interest.

## Supporting information

Supporting Information

Supporting Information

Supporting Information

Supporting Information

Supporting Information

Supporting Information
