# Supplementary figures and images for: Comprehensive mapping of B lymphocyte immune dysfunction in idiopathic nephrotic syndrome children
Source: Clin Transl Med. 2023 Feb 5;13(2):e1177. doi: 10.1002/ctm2.1177 (PMC9899684; doi:10.1002/ctm2.1177)

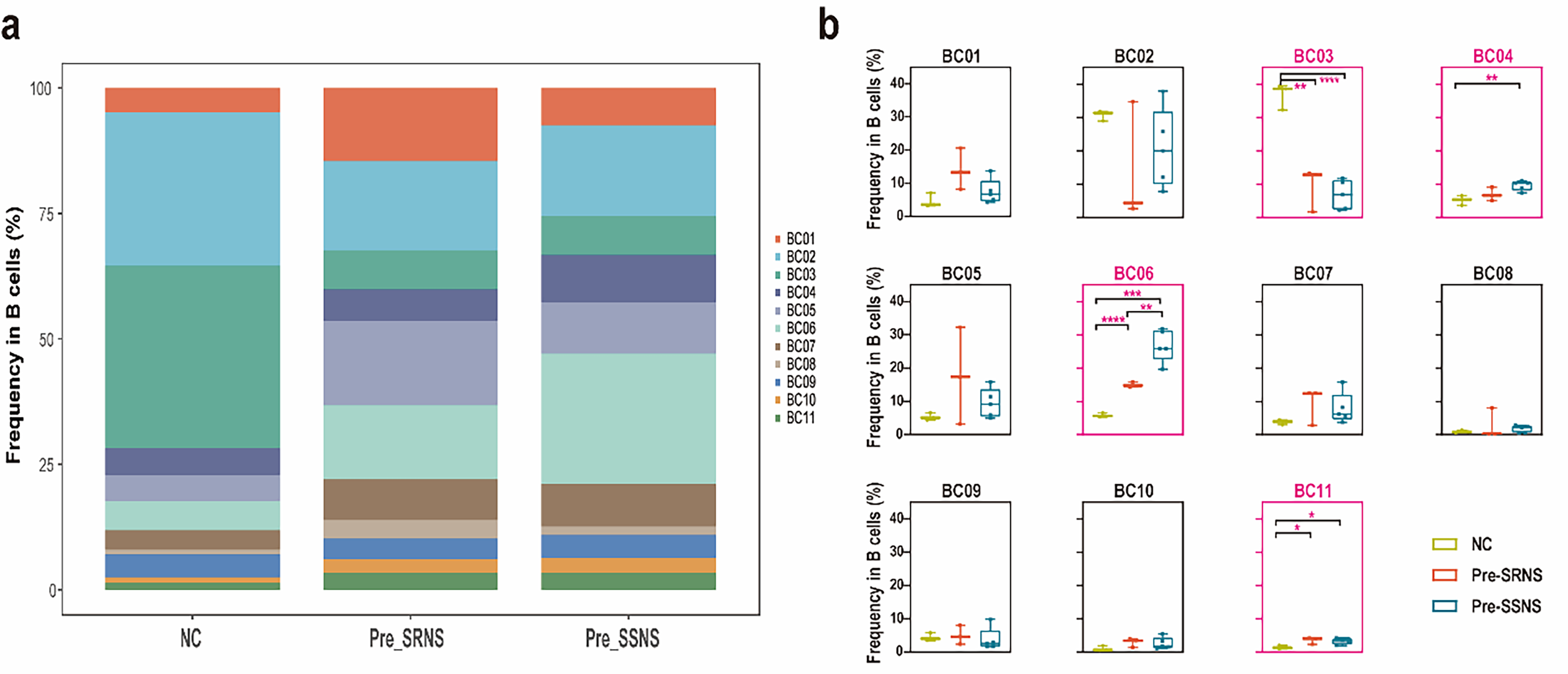

Supplement: Supplementary file 1 — Supporting Information [file CTM2-13-e1177-s006.tif]

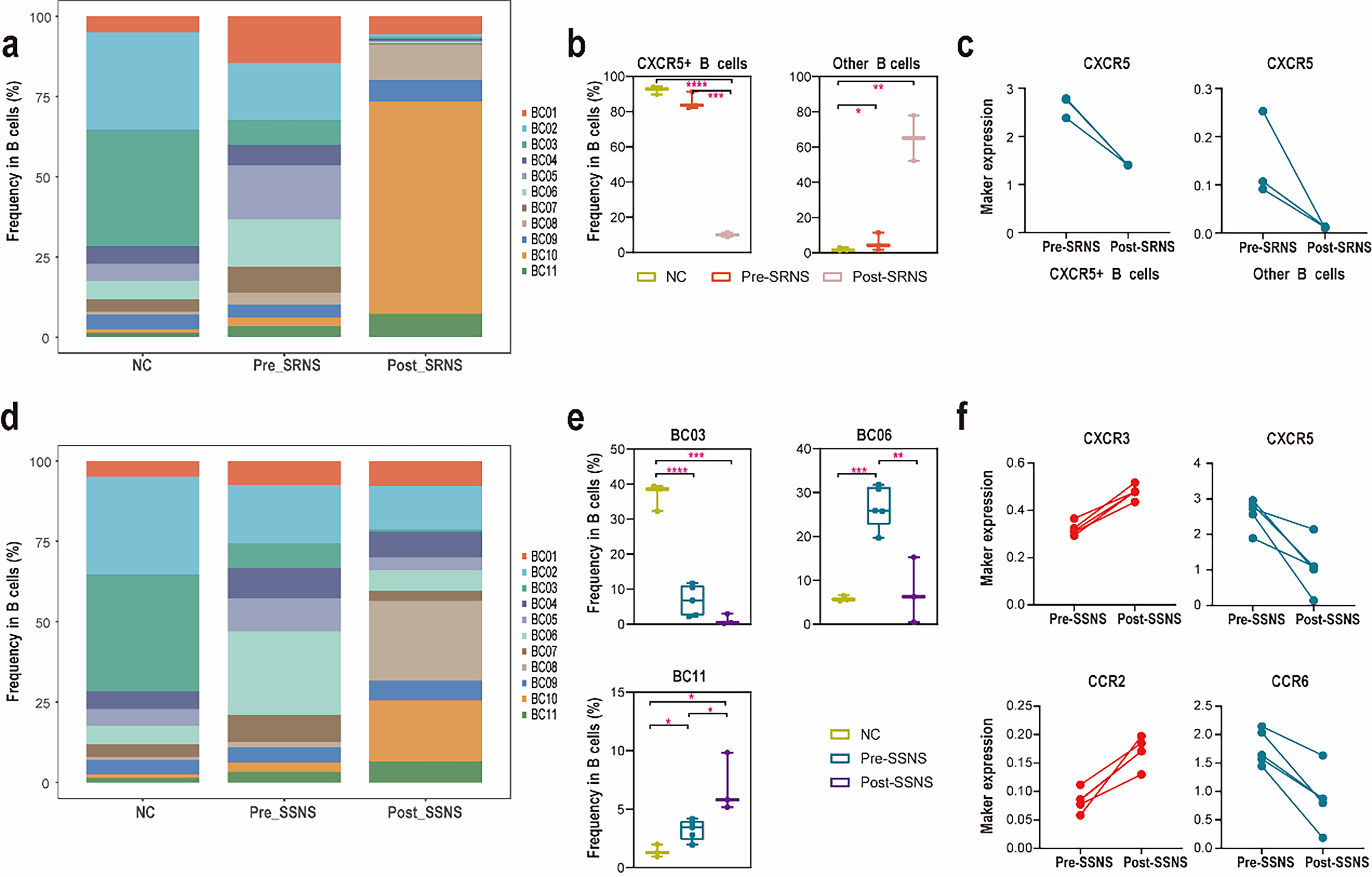

Supplement: Supplementary file 2 — Supporting Information [file CTM2-13-e1177-s003.tif]

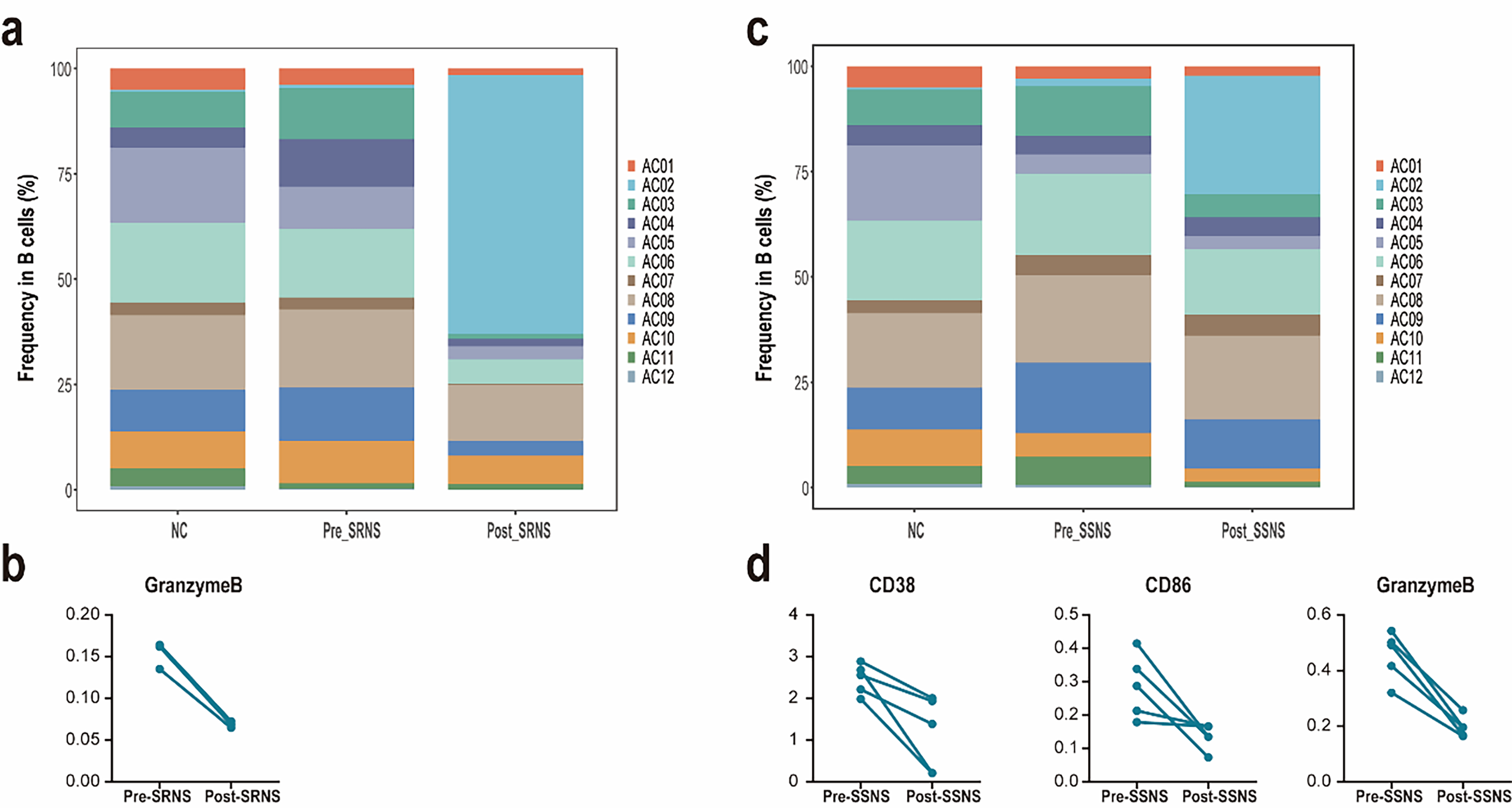

Supplement: Supplementary file 3 — Supporting Information [file CTM2-13-e1177-s004.tif]
