# Supplementary material for: Comprehensive mapping of B lymphocyte immune dysfunction in idiopathic nephrotic syndrome children
Source: Clin Transl Med. 2023 Feb 5;13(2):e1177. doi: 10.1002/ctm2.1177 (PMC9899684; doi:10.1002/ctm2.1177)
Supplement: Supplementary file 4 — Supporting Information [file CTM2-13-e1177-s002.docx]

Supplementary Table 1. Characteristics of subjects in the present study

| Patient | Gender | Age | Steroid responsiveness | Pathology | Treatment |
| --- | --- | --- | --- | --- | --- |
| LS | M | 10y | SRNS | MCD | Tacrolimus |
| LCX | M | 13y9m | SRNS | MsPGN | Tacrolimus |
| YMY  WLW  HYX  CZY  JSH  CZY  NC-1  NC-2  NC-3 | M  F  M  M  F  F  M  M  F | 8y11m  6y1m  8y6m  4y4m  7y7m  5y2m  6y10m  10y9m  4y5m | SRNS  SSNS  SSNS  SSNS  SSNS  SSNS  /  /  / | FSGS  /  /  /  /  MCD  /  /  / | Tacrolimus  Steroid  Steroid  Steroid  Steroid  Steroid  /  /  / |

NC, normal control; SSNS, steroid-sensitive nephrotic syndrome; SRNS, steroid-resistant; MCD, minimal change disease; MsPGN, mesangial proliferative glomerulonephritis; FSGS, focal segmental glomerulosclerosis.
