# Supplementary material for: Comprehensive mapping of B lymphocyte immune dysfunction in idiopathic nephrotic syndrome children
Source: Clin Transl Med. 2023 Feb 5;13(2):e1177. doi: 10.1002/ctm2.1177 (PMC9899684; doi:10.1002/ctm2.1177)
Supplement: Supplementary file 5 — Supporting Information [file CTM2-13-e1177-s001.docx]

**Supplementary Table 2. Each marker annotation of Panel A and Panel B.**

| **Panel A** | | **Panel B** | |
| --- | --- | --- | --- |
| **Ag** | **Description** | **Ag** | **Description** |
| **Immunomodulatory molecules** | | **Immunomodulatory molecules** | |
| CD27 | Costimulatory Molecules | CD152(CTLA-4) | Inhibitory Molecules |
| CD278(ICOS) | Costimulatory Molecules | CD279(PD-1) | Inhibitory Molecules |
| CD69 | Costimulatory Molecules | CD161 | stimulatory receptor |
| CD57 | Costimulatory Molecules | CD103 | Adhesion Molecules |
| CD314(NKG2D) | Costimulatory Molecules | CD107a | Adhesion Molecules |
| CD152(CTLA-4) | Inhibitory Molecules | CD57 | Costimulatory Molecules |
| CD159a(NKG2A) | Inhibiting NK-cell receptor | **Immune context** | |
| CD279(PD-1) | Inhibitory Molecules | CD45 | Pan Immune |
| CD33 | Adhesion Molecules | CD3 | T cells |
| CD303 | Adhesion Molecules | CD56 | NK cells |
| CD38 | Adhesion Molecules | TCRγδ | γδT cells |
| HLA-DR | Antigen-presenting cells | CD14 | Monocytes |
| CD1c | APC | CD19 | B cells |
| CD141 | Thrombin | CD68 | myeloid cells |
| CD39 | Activated lymphocytes | CD206 | M2 |
| **Immune context** | | CD4 | CD4T |
| CD45 | Pan Immune | CD8 | CD8 T |
| CD3 | T cells | CD11b | myeloid cells |
| CD66b | Granulocytes | CD16 | Monocytes |
| CD56 | NK cells | CD86 | M1 |
| gdTCR | γδT cells | CD45RA | Naive T cells |
| CD14 | Monocytes | **Cytokines** | |
| CD123 | pDC or Basophils | IFN-r | Immune interferon |
| CD19 | B cells | IL-4 | Interleukin-4 |
| CD68 | myeloid cells | TNF-a | Tumor necrosis factor-α |
| CD11c | DCs | IL-2 | Interleukin-2 |
| CD16 | Monocytes | IL-22 | Interleukin-22 |
| CD4 | CD4 T | IL-6 | Interleukin-6 |
| CD8 | CD8 T | IL-10 | Interleukin-10 |
| CD11b | myeloid cells | IL-17A | Interleukin-17A |
| CD163 | M2 | IL-8 | Interleukin-8 |
| CD86 | M1 | IL-23 | Interleukin-23 |
| CD45RA | Naive T cells | IL-1b(IL-1F2) | Interleukin-1β |
| FoxP3 | Treg | IL-17F | Interleukin-17F |
| CD25(IL-2Ra) | Treg | GM-CSF | Granulocyte/macrophage colony-stimulating factor |
| **Cyto-/chemokine receptor** | | IL-9 | Interleukin-9 |
| CD196(CCR6) | Chemokine Receptors | TGF-b1(LAP) | Latency-associated peptide |
| CD183(CXCR3) | Chemokine Receptors | Granzyme B | CTLS |
| CD194(CCR4) | Chemokine Receptors | **Cyto-/chemokine receptor** | |
| CD197(CCR7) | Chemokine Receptors | CD196(CCR6) | Chemokine Receptors |
| CD185(CXCR5) | Chemokine Receptors | CD183(CXCR3) | Chemokine Receptors |
| CD25(IL-2Ra) | Cytokine Receptors | CD195(CCR5) | Chemokine Receptors |
| CD127 | Cytokine Receptors | CD192(CCR2) | Chemokine Receptors |
| **Intracellular/Transcription Factors** | | CD185(CXCR5) | Chemokine Receptors |
| FoxP3 | Forkhead/winged-helix transcription factor | CD25(IL-2Ra) | Cytokine Receptors |
| T-bet | T-box transcription factor | / | / |
| Granzyme B | Cytotoxic T-lymphocyte-associated serine esterase 1 | / | / |
