# Supplementary material for: Comprehensive mapping of B lymphocyte immune dysfunction in idiopathic nephrotic syndrome children
Source: Clin Transl Med. 2023 Feb 5;13(2):e1177. doi: 10.1002/ctm2.1177 (PMC9899684; doi:10.1002/ctm2.1177)
Supplement: Supplementary file 6 — Supporting Information [file CTM2-13-e1177-s005.docx]

**Supplementary Table 3. Lists of purified antibodies in mass cytometry analysis**

**Panel A**

| **List** | ***** **Metal channel** | ***** **Antibody** | ***Clone** | **Vender** | **Cat#** |
| --- | --- | --- | --- | --- | --- |
| 1 | **89Y** | **CD45** | **HI30** | BioLegend | 304002 |
| 2 | **115In** | **CD3** | **UCHT1** | BioLegend | 300438 |
| 3 | **139La** | **CD66b** | **G10F5** | BioLegend | 305102 |
| 4 | **141Pr** | **CD56** | **NCAM16.2** | BD | 559043 |
| 5 | **142Nd** | **gdTCR** | **5A6.E9** | PLT | |
| 6 | **143Nd** | **CD196_CCR6** | **G034E3** | BioLegend | 353402 |
| 7 | **144Nd** | **CD14** | **M5E2** | BioLegend | 301810 |
| 8 | **145Nd** | **CD27** | **O323** | BioLegend | 302802 |
| 9 | **146Nd** | **CD123** | **6H6** | BioLegend | 306002 |
| 10 | **147Sm** | **CD183_CXCR3** | **G025H7** | BioLegend | 353750 |
| 11 | **148Nd** | **CD19** | **HIB19** | BioLegend | 302214 |
| 12 | **149Sm** | **CD25_IL_2Ra** | **24212** | RD | MAB1020 |
| 13 | **150Nd** | **CD1c** | **L161** | BioLegend | 331502 |
| 14 | **151Eu** | **CD278_ICOS** | **C398.4A** | BioLegend | 313502 |
| 15 | **152Sm** | **CD39** | **A1** | BioLegend | 328202 |
| 16 | **153Eu** | **CD57** | **HCD57** | BioLegend | 322325 |
| 17 | **154Sm** | **CD163** | **GHI/61** | BioLegend | 333602 |
| 18 | **155Gd** | **CD45RA** | **HI100** | BioLegend | 304102 |
| 19 | **156Gd** | **CD194_CCR4** | **L291H4** | BioLegend | 359402 |
| 20 | **157Gd** | **CD68** | **Y1/82A** | BioLegend | 333802 |
| 21 | **158Gd** | **CD86** | **Fun-1** | BD | 555655 |
| 22 | **159Tb** | **CD11c** | **BU15** | BioLegend | 337202 |
| 23 | **160Gd** | **CD33** | **WM53** | BioLegend | 303419 |
| 24 | **161dy** | **CD152_CTLA_4** | **14D3** | eB | 14-1529-82 |
| 25 | **162Dy** | **FoxP3** | **PCH101** | eB | 14-4776-82 |
| 26 | **163Dy** | **CD159a_NKG2A** | **131411** | RD | MAB1059 |
| 27 | **164Dy** | **CD141** | **M80** | BioLegend | 344102 |
| 28 | **165Ho** | **CD303** | **201A** | Biolegend | 354215 |
| 29 | **166Er** | **CD314_NKG2D** | **1D11** | BioLegend | 320814 |
| 30 | **167Er** | **CD197_CCR7** | **G043H7** | BioLegend | 353222 |
| 31 | **168Er** | **T-bet** | **4B10** | BioLegend | 644802 |
| 32 | **169Tm** | **CD185_CXCR5** | **RF8B2** | BD | 552032 |
| 33 | **170Er** | **CD127** | **A019D5** | BioLegend | 351302 |
| 34 | **171Yb** | **CD69** | **FN50** | BioLegend | 310902 |
| 35 | **172Yb** | **CD38** | **HIT2** | BioLegend | 303502 |
| 36 | **173Yb** | **GranzymeB** | **QA16A02** | BioLegend | 372202 |
| 37 | **174Yb** | **CD279_PD_1** | **EH12.2H7** | BioLegend | 329926 |
| 38 | **175Lu** | **CD16** | **3G8** | BioLegend | 302014 |
| 39 | **176Yb** | **HLA_DR** | **L243** | BioLegend | 307612 |
| 40 | **197Au** | **CD4** | **RPA-T4** | BioLegend | 300516 |
| 41 | **198Pt** | **CD8** | **RPA-T8** | BioLegend | 301018 |
| 42 | **209Bi** | **CD11b** | **M1/70** | BioLegend | 101202 |

Panel B

| **List** | ***** **Metal channel** | ***** **Antibody** | ***Clone** | **Vender** | **Cat#** |
| --- | --- | --- | --- | --- | --- |
| **1** | **89Y** | **CD45** | **HI30** | BioLegend | 304002 |
| **2** | **115In** | **CD3** | **UCHT1** | BioLegend | 300438 |
| **3** | **139La** | **IFN_r** | **B27** | BioLegend | 506502 |
| **4** | **141Pr** | **CD56** | **NCAM16.2** | BD | 559043 |
| **5** | **142Nd** | **gdTCR** | **5A6.E9** | PLT | |
| **6** | **143Nd** | **CD196_CCR6** | **G034E3** | BioLegend | 353402 |
| **7** | **144Nd** | **CD14** | **M5E2** | BioLegend | 301810 |
| **8** | **145Nd** | **IL_4** | **MP4-25D2** | BioLegend | 500802 |
| **9** | **146Nd** | **TNF_a** | **Mab11** | BioLegend | 502902 |
| **10** | **147Sm** | **CD183_CXCR3** | **G025H7** | BioLegend | 353750 |
| **11** | **148Nd** | **IL-2** | **MQ1-17H12** | BioLegend | 500302 |
| **12** | **149Sm** | **CD25_IL_2Ra** | **24212** | RD | MAB1020 |
| **13** | **150Nd** | **IL-22** | **22URTI** | Invitrogen | 50-7229-42 |
| **14** | **151Eu** | **CD107a** | **H4A3** | BioLegend | 328602 |
| **15** | **152Sm** | **CD195_CCR5** | **J418F5** | BioLegend | 359102 |
| **16** | **153Eu** | **CD57** | **HCD57** | BioLegend | 322325 |
| **17** | **154Sm** | **CD152_CTLA_4** | **14D3** | eB | 14-1529-82 |
| **18** | **155Gd** | **CD45RA** | **HI100** | BioLegend | 304102 |
| **19** | **156Gd** | **IL_6** | **MQ2-13A5** | BioLegend | 501102 |
| **20** | **157Gd** | **IL_10** | **JES3-9D7** | BioLegend | 501402 |
| **21** | **158Gd** | **CD19** | **HIB19** | BioLegend | 302214 |
| **22** | **159Tb** | **IL_17A** | **BL168** | BioLegend | 512302 |
| **23** | **160Gd** | **IL_8** | **E8N1** | BioLegend | 511402 |
| **24** | **161Dy** | **IL_23** | **23dcdp** | Invitrogen | 50-7823-42 |
| **25** | **162Dy** | **IL_1b_IL_1F2** | **8516** | RD | MAB201-500 |
| **26** | **163Dy** | **CD68** | **Y1/82A** | BioLegend | 333802 |
| **27** | **164Dy** | **CD192_CCR2** | **K036C2** | BioLegend | 357202 |
| **28** | **165Ho** | **CD161** | **HP-3G10** | BioLegend | 339902 |
| **29** | **166Er** | **IL_17F** | **SHLR17** | Invitrogen | 16-7169-82 |
| **30** | **167Er** | **CD206** | **15-2** | BioLegend | 321112 |
| **31** | **168Er** | **GM_CSF** | **BVD2-21C11** | BioLegend | 502302 |
| **32** | **169Tm** | **CD185_CXCR5** | **RF8B2** | BD | 552032 |
| **33** | **170Er** | **CD86** | **Fun-1** | BD | 555655 |
| **34** | **171Yb** | **IL_9** | **MH9A4** | Invitrogen | 50-7097-42 |
| **35** | **172Yb** | **CD279_PD_1** | **EH12.2H7** | BioLegend | 329926 |
| **36** | **173Yb** | **Granzyme B** | **QA16A02** | BioLegend | 372202 |
| **37** | **174Yb** | **TGF_b1_LAP** | **TW4-6H10** | BioLegend | 349702 |
| **38** | **175Lu** | **CD16** | **3G8** | BioLegend | 302014 |
| **39** | **176Yb** | **CD103** | **B-Ly7** | eB | 14-1038-82 |
| **40** | **197Au** | **CD4** | **RPA-T4** | BioLegend | 300516 |
| **41** | **198Pt** | **CD8** | **RPA-T8** | BioLegend | 301018 |
| **42** | **209Bi** | **CD11b** | **M1/70** | BioLegend | 101202 |
